# The association of herpes zoster and influenza vaccinations with the risk of developing dementia: a population-based cohort study within the UK Clinical Practice Research Datalink

**DOI:** 10.1186/s12889-023-16768-4

**Published:** 2023-10-02

**Authors:** Artitaya Lophatananon, Matthew Carr, Brian Mcmillan, Curtis Dobson, Ruth Itzhaki, Rosa Parisi, Darren M. Ashcroft, Kenneth R. Muir

**Affiliations:** 1 Division of Population Health, Health Services Research and Primary Care, School of Health Sciences, Faculty of Biology, Medicine and Health, The University of Manchester, Manchester, M13 9PT UK; 2 Centre for Pharmacoepidemiology and Drug Safety, Division of Pharmacy & Optometry, School of Health Sciences, Faculty of Biology, Medicine and Health, The University of Manchester, M13 9PT Manchester, UK; 3 NIHR Greater Manchester Patient Safety Translational Research Centre (PSTRC), School of Health Sciences, Faculty of Biology, Medicine and Health, The University of Manchester, Manchester, M13 9PT UK; 4 The Oxford Institute of Population Ageing, University of Oxford, Oxford, OX2 6PR UK; 5 Division of Informatics, Imaging & Data Sciences, School of Health Sciences, Faculty of Biology, Medicine and Health, The University of Manchester, Manchester, M13 9PT UK

**Keywords:** Zoster vaccination, Dementia

## Abstract

**Background:**

Dementia affects ability to remember, think, or make decisions that interfere with doing everyday activities. There is no cure, therefore any prevention or delay of the onset is of importance. This study aims to investigate the association between zoster and influenza vaccinations and the risk of developing dementia.

**Methods:**

We conducted a retrospective population-based cohort study using electronic health records from 1469 general practices contributing to the Clinical Practice Research Datalink (CPRD) *Aurum* database with linked hospital episode statistics (HES) and Office for National Statistics (ONS) mortality records. We built two 'matched cohorts': zoster vaccine (854,745 exposed individuals) matched with 8.8 million comparators without a history of zoster vaccination, and influenza vaccine (742,487 exposed individuals) matched with 7.12 million comparators without a history of vaccination as another comparator group. The cohorts were then followed to assess the association of exposure (vaccine) with outcome (dementia diagnosis).

**Results:**

Zoster vaccination was associated with a lower risk of dementia diagnosis (adjusted hazard ratio (HR) 0.78 with 95% CI: 0.77–0.79), Alzheimer’s diagnosis (adjusted HR 0.91 with 95% CI: 0.89–0.92 and other types of dementia (adjusted HR 0.71 with 95% CI: 0.69–0.72). Influenza vaccination also was associated with a slightly reduced hazard of dementia risk (adjusted HR 0.96 with 95% CI: 0.94–0.97).

**Conclusion:**

Both zoster vaccine for prevention of shingles / herpes zoster and influenza vaccine to prevent influenza were associated with diminished risk of dementia, with the zoster association appearing more pronounced.

**Supplementary Information:**

The online version contains supplementary material available at 10.1186/s12889-023-16768-4.

## Background

Globally, it has been estimated that the number of people with dementia would increase from 57.4 million cases in 2019 to 152.8 (130.8–175.9) million cases in 2050 [1]. Dementia affects 850,000 people in the UK. It has been estimated to rise to one million by 2025. The National Health Service costs for its management are also expected to double by 2050 [2]. In addition to lifestyle and genetic factors, epidemiological studies have linked infection by various types of virus with dementia [3–7]. In recent years, several studies have investigated the effect of herpes zoster (HZ also known as shingles) on dementia risk [8–14], and in 2021, we reported a reduction between zoster vaccination and dementia risk using data from the UK Biobank study (OR: 0.81 with 95% CI: 0.66 to 0.99) [12]. Also in 2021, a study analysed medical health record data in a large Veterans Health Affairs (VHA) cohort and replicated their analysis in another cohort comprisingMarketScan® commercial and Medicare claims. The authors reported a significant association with lower dementia risk in both cohorts (VHA HR = 0.69; 95% CI: 0.67–0.72; MarketScan HR = 0.65; 95% CI: 0.57–0.74) [14]. Another recent study used data from The Behavioral Risk Factor Surveillance System (BRFSS) to evaluate the relationship between HZ vaccination and cognitive impairment. They also reported that herpes zoster vaccination reduces the risk of dementia [15]. In 2022, a meta-analysis and systematic review of seven studies with 1,857,134 participants from population-based observational studies reported overall pooled results indicating that several common types of vaccine, including those against HZ and influenza were associated with lower dementia risk (HR = 0.65, 95% CI: 0.60–0.71, *P*-value overall effect < 0.001; I^2^ = 91.8%, *P*-value heterogeneity < 0.001) [16]. Questions remain as to whether residual confounding is affecting all of these observational studies and also as to whether there is a specific effect of vaccinations against herpes zoster or a non-specific immune stimulatory effect of multiple vaccinations. It is against this background that we sought to explore the separate effects of both zoster and flu vaccination in a large nationally representative dataset with time dependent adjustment for potential confounders including that of prescribed anti-viral medications.

In the UK, shingles vaccination has been offered routinely by the National Health Service (NHS) since 2013 for people aged 70–80 years. In this study, we aimed to investigate the association between zoster vaccination for shingles and dementia risk using data from the Clinical Practice Research Datalink (CPRD) *Aurum* database. As influenza (flu) vaccination is also common in the UK, we explored the association between influenza vaccination and dementia risk, which we considered to represent a comparator.

## Methodology

### Data sources

We conducted retrospective matched cohort studies using primary care electronic health records from the Clinical Practice Research Datalink (CPRD) *Aurum* database obtained under licence from the UK Medicines and Healthcare products Regulatory Agency (MHRA) [17, 18]. The CPRD contains anonymised consultation records and includes patient demographic information, symptoms, diagnoses, medication prescriptions, and vaccination data. In June 2021, there were 1360 general practices using the EMIS clinical platform that were based in England and contributing data to CPRD *Aurum* [19]. We restricted our cohorts to patients from these practices as data linkage is only available for English practices that participate in the CPRD linkage scheme, linking data for all eligible patients with a valid National Health Service (NHS) identifier. We utilised linkages between CPRD *Aurum* and external data sources. Inpatient Hospital Episode Statistics (HES) were used to augment capture of diagnoses of dementia (as both an outcome and an exclusion factor) and comorbidities. Data from the Office for National Statistics (ONS) were used to ascertain specific causes of death according to the 10th revision of the International Classification of Diseases (ICD-10). Individual patients are entitled to opt out of the linkage scheme.

### Study cohorts

Cohort members were aged 70 years and over, were registered in a practice contributing to CPRD *Aurum*, and were administered their first zoster vaccination between 1^st^ January 2013 and 31^st^ October 2020 (identified using the product codes listed in Additional file 1 and the clinical Read/SNOMED/EMIS codes listed in Additional file 2). We did not include the Shingrix vaccine because it was not in use in the contributing CPRD practices during our study period (the end of the study period was 31st October 2020). Henceforth, we define a patient’s index date as the date of the first recorded evidence that a relevant vaccination occurred. We included patients who had been registered with their general practice for at least one year and had no recorded history of dementia at that time. Follow-up ended on the first to occur of dementia diagnosis (in primary or secondary care); death; migration from general practice; the end of data collection for the practice; the end of the study period (31^st^ October 2020).

Using incidence density sampling, a representative comparison cohort was created whereby each patient with an incident vaccination was matched with up to 10 comparators without a history of zoster vaccination to create a ‘matched set’. Requiring the comparators to have been unaffected by any form of dementia prior to the index date of the 'case', we matched the patients on age, gender, and registered general practice. The same registration and practice contribution criteria were applied when sampling patients for the comparison cohort. The process of matching was as follows. Each vaccinated patient was matched on sex, age (within 3 years), and general practice with up to 10 registered comparators on the date of vaccination. The comparators were required to have no history of the vaccination in question. Matching was undertaken using the nearest neighbour approach (via the minimisation of the Mahalanobis distance [20]). In the event of ties, random selection was used. The distribution of the number of comparators in the matched sets can be found in Additional file 3. To update vaccine status, patients who featured as comparators (without a history of the relevant vaccination type) could subsequently go on to become a case if they received the vaccine at a later date. At this point, their follow-up time as a comparator would be terminated. The influenza vaccination cohort was delineated in similar way to zoster vaccination cohort. Influenza vaccine identified using the product codes listed in Additional file 4 and the clinical Read/SNOMED/EMIS codes listed in Additional file 5. In this study, repeat vaccinations are not incorporated. Once a patient has a relevant vaccine in their record, they are defined as a ‘case’ and their exposure status is described using a dichotomous variable: “Ever exposed to zoster vaccine? = YES”.

### Dementia diagnoses

Diagnoses of dementia were identified in the primary care records using the Read/SNOMED/EMIS codes listed in Additional file 6, and in the HES and ONS mortality records using the International Classification of Diseases 10th Revision (ICD-10) codes listed in Additional file 7. We also looked separately at diagnoses of Alzheimer’s disease (AD) and other forms of dementia (an identifier is included in the code lists). Dementia diagnosis dates were used as both the study outcome and for exclusions from the study cohort if the diagnosis was recorded prior to vaccination or matching (for the comparison group).

### Statistical analyses

All data processing and statistical analyses were conducted using Stata V.16 (StataCorp, College Station, Texas, USA). To compare the prevalence of comorbidities at baseline in the vaccinated patients and their matched comparators, we used conditional Poisson regression. For the specific forms of dementia (Alzheimer’s disease and 'other' dementia) and for the composite outcome ('any dementia'), we estimated the relative risk of onset using Cox regression models with stratification on the matched sets of exposed individuals and comparators. The proportional hazards assumption was formally assessed using the Grambsch–Therneau test [21] and graphical diagnostics were performed using scaled Schoenfeld residuals [22]. Stratified analyses were conducted according to gender and age-band.

By virtue of the matched cohort study design, the estimated hazard ratios (HRs) were naturally adjusted for the potentially confounding effects of age, gender, and practice-level factors. We also applied a series of adjustments on patient-level socio-economic status, ethnicity, indicators for the conditions included in the Charlson Comorbidity Index (CCI) (treated in separate models as baseline comorbidities and as time-dependent confounders) [23], and varicella-zoster antiviral status (again, treated as a baseline risk factor and as a time-dependent covariate in separate models). Read/SNOMED/EMIS codes used to identify the comorbidities included in the CCI can be found in Additional file 8. The corresponding ICD-10 codes can be found in Additional file 9. The codes for antiviral treatments can be found in Additional file 10.

Patient-level socioeconomic status was measured, according to patient postcodes, using English Indices of Multiple Deprivation (IMD) quintiles [24], a measure representing an area’s relative level of deprivation, ranked within England. Ethnicity was classified from primary care and Hospital Episode Statistics (HES) records as white/Asian/black/mixed/other. The relevant Read/SNOMED/EMIS code list can be found in Additional file 11. For patients with multiple records and conflicts, we defined ethnicity as (a) the category with most corresponding records; (b) according to the last available record when record numbers were tied across two or more categories; and (c) by randomly selecting between candidate ethnicities when record numbers were tied across two or more categories and multiple categories were entered in the last available record.

We fitted 3 different models.

- Model 1: Adjusted for age, gender, and general practice factors (based on matched design).
- Model 2: Further adjustments for IMD, ethnicity, and time-dependent indicators for the conditions in the Charlson Comorbidity Index.
- Model 3: Further adjustments for IMD, ethnicity, time-dependent indicators for the conditions in the Charlson Comorbidity Index, and time-dependent antiviral status.

### Sensitivity analyses

We used the E-value to demonstrate the minimum strength of association that an unmeasured confounder would need to have with both the exposure and the outcome to fully explain away a specific exposure–outcome association, conditional on the measured covariates. The E-value can be calculated for an observed risk ratio (denoted RR) by E-value = $RR+\sqrt{RR\times RR-1}$| If the original risk ratio is below 1, then the inverse is taken before applying the E-value formula. This formula can also be used for hazard ratio [25]. The lowest possible E-value is 1 (i.e. no unmeasured confounding is needed to explain away the observed association). The higher the E-value the stronger the confounder associations would have to be to explain away the effect [26]. Finally, we took a further step to assess the potential for residual unmeasured confounding of our exposure–outcome association measures, by conducting a set of comparator analyses with influenza vaccination as the exposure of interest. All aspects of the analyses were identical with the exception that the index dates (and subsequent delineation of the matched cohort) were defined by vaccination for influenza, rather than zoster vaccination.

We also carried out sensitivity analysis with left-censoring at 2 years after vaccination (or matching for the comparators) to assess the estimated HRs for a longer period after vaccination.

#### Patient and public involvement

There was no patient or public involvement in the study including development of the research question, selection of outcome measures, study design, conduct or dissemination of findings.

## Results

Table 1 shows the characteristics for both the zoster and influenza vaccine cohorts. The total number of participants in the zoster vaccinated group was 854,745 and approximately 8.8 million participants with no record of zoster vaccination. The total number in the influenza vaccinated group was 742,487 and approximately 7.12 million participants with no record of influenza vaccination. The numbers of people developing dementia in zoster vaccinated and unvaccinated cohorts were 28,012 with a rate of 9.9 per 1000 person-year (95% CI 9.8–10.0) and 197,618 with a rate of 12.1 per 1000 person-year (95% CI 12.1–12.2), respectively. The numbers of people developing dementia in influenza vaccinated and unvaccinated cohorts were 21,931 with a rate of 17.2 per 1000 person-year (95%CI 17.0–17.4) and 120,297 with a rate of 15.8 (95%CI 15.7–15.9). Results can be found in Additional file 12. In both cohorts, the majority of participants are in the age group 70–74 and of white ethnicity (~ 80%). There was also a slightly higher number of females than males in both cohorts. Around a quarter of participants were from areas in the least deprived quintile. The distribution of IMD was similar between zoster and influenza vaccination cohorts. The highest prevalence ratio of co-morbidity in both zoster and influenza cohort – comparison of the prevalence between 'vaccinated group' VS 'comparator (non-vaccinated) group’—was diabetes with chronic complications (1.12 and 1.19). Similar results were seen with anti-viral treatments (1.06 for the zoster cohort and 1.10 for the influenza cohort). The median follow-up times in the zoster vaccine exposed individuals and the unvaccinated comparator group were 3.07 yrs (IQR: 1.73, 5.04) and 3.01 yrs (IQR: 1.57, 4.99). The median follow-up times in the influenza vaccine exposed individuals and the unvaccinated comparator group were 1.08 yrs (IQR: 0.98, 2.07) and 1.08 yrs (IQR: 0.97, 2.07). Table 2 showed the hazard ratio of developing dementia and subtypes with zoster vaccination as an exposure. Results from all models suggested zoster vaccination was significantly associated with a reduced risk of developing dementia. The fully adjusted model (model 3: adjusted for IMD, ethnicity, time-dependent indicators for the conditions in the Charlson Comorbidity Index, and time-dependent antiviral status) produced a hazard ratio of 0.78 (with 95% CI: 0.77–0.79) and for other types of dementia (HR: 0.71; 95% CI: 0.69–0.72). The reduced HR was less pronounced in AD (HR: 0.91; 95% CI: 0.89–0.92). The results from the stratified analysis by gender suggested similar patterns as seen in non-stratified analysis. However, the HRs for males were slightly higher than those for females. The HRs for all types of dementia decreased with increasing age group.

**Table 1:** General characteristics of vaccinated and unvaccinated groups based on vaccination type

Vaccination type	Demographic/Characteristic		Vaccinated patients		Matched comparators		Prevalence ratio (95% CI)
			***n***	**%**	***n***	**%**	
Zoster	All		854,745		8,490,813		
	Gender	Male	407,361	47.7	4,046,215	47.7	
		Female	447,384	52.3	4,444,598	52.3	
	Age	70–74	519,822	60.8	5,177,036	61.0	
		75–79	275,698	32.3	2,733,974	32.2	
		80–84	58,401	6.8	572,024	6.7	
		85–89	610	0.1	5,821	0.1	
		90 +	214	0.03	1,958	0.02	
	Ethnicity	White	702,463	82.2	6,636,714	78.2	
		Asian	25,113	2.9	226,362	2.7	
		Black	10,488	1.2	116,742	1.4	
		Other	8,569	1.0	93,022	1.1	
		Unknown	108,112	12.6	1,417,973	16.7	
	IMD	1 (least deprived)	236,851	27.7	2,238,248	26.4	
		2	205,189	24.0	2,000,088	23.6	
		3	171,309	20.0	1,721,476	20.3	
		4	137,555	16.1	1,412,409	16.6	
		5 (most deprived)	103,292	12.1	1,112,569	13.1	
		Unknown	549	0.1	6,023	0.1	
	Baseline comorbidities:						
		Any malignancy	193,171	22.6	1,905,820	22.4	1.01 (1.00, 1.01)
		Cerebrovascular disease	85,038	9.9	858,129	10.1	0.98 (0.98, 0.99)
		Chronic pulmonary disease	171,459	20.1	1,587,020	18.7	1.07 (1.07, 1.08)
		Congestive heart failure	44,400	5.2	454,834	5.4	0.97 (0.96, 0.98)
		Diabetes with chronic complications	103,028	12.1	909,926	10.7	1.12 (1.12, 1.13)
		Diabetes without chronic complications	161,741	18.9	1,443,053	17.0	1.11 (1.11, 1.12)
		AIDS/HIV	194	0.02	4,855	0.1	0.40 (0.34, 0.46)
		Hemiplegia or paraplegia	8,184	1.0	105,649	1.2	0.77 (0.75, 0.79)
		Metastatic solid tumour	14,187	1.7	195,887	2.3	0.72 (0.71, 0.73)
		Mild liver disease	14,457	1.7	160,340	1.9	0.90 (0.88, 0.91)
		Moderate or severe liver disease	5,505	0.6	65,993	0.8	0.83 (0.81, 0.85)
		Myocardial infarction	61,399	7.2	588,844	6.9	1.03 (1.03, 1.04)
		Peptic ulcer disease	59,548	7.0	571,816	6.7	1.03 (1.02, 1.04)
		Peripheral vascular disease	50,170	5.9	502,301	5.9	0.99 (0.98, 1.00)
		Renal disease	143,735	16.8	1,371,452	16.2	1.04 (1.03, 1.05)
		Rheumatic disease	55,879	6.5	653,749	7.7	0.85 (0.84, 0.86)
	Antiviral treatment history (at baseline)		90,344	10.6	845,499	10.0	1.06 (1.05, 1.07)
Influenza	All		742,487		7,219,770		
	Gender	Male	344,460	46.4	3,351,283	46.4	
		Female	398,027	53.6	3,868,487	53.6	
	Age	70–74	307,303	41.4	3,071,413	42.5	
		75–79	201,955	27.2	1,992,571	27.6	
		80–84	130,963	17.6	1,271,165	17.6	
		85–89	68,996	9.3	640,653	8.9	
		90 +	33,270	4.5	243,968	3.4	
	Ethnicity	White	607,439	81.8	5,468,741	75.7	
		Asian	21,003	2.8	167,049	2.3	
		Black	9,630	1.3	106,665	1.5	
		Other	7,763	1.0	75,869	1.1	
		Unknown	96,652	13.0	1,401,446	19.4	
	IMD	1 (least deprived)	199,952	26.9	1,877,787	26.0	
		2	172,867	23.3	1,664,335	23.1	
		3	149,466	20.1	1,460,983	20.2	
		4	122,125	16.4	1,204,077	16.7	
		5 (most deprived)	97,481	13.1	1,005,699	13.9	
		Unknown	596	0.1	6,889	0.1	
	Baseline comorbidities:						
		Any malignancy	201,889	27.2	1,730,019	24.0	1.13 (1.12, 1.13)
		Cerebrovascular disease	98,482	13.3	880,249	12.2	1.07 (1.06, 1.08)
		Chronic pulmonary disease	154,449	20.8	1,254,053	17.4	1.19 (1.19, 1.20)
		Congestive heart failure	57,757	7.8	485,843	6.7	1.13 (1.12, 1.14)
		Diabetes with chronic complications	91,204	12.3	742,247	10.3	1.19 (1.18, 1.20)
		Diabetes without chronic complications	141,673	19.1	1,176,490	16.3	1.17 (1.16, 1.17)
		AIDS/HIV	277	0.04	2,564	0.04	1.06 (0.93, 1.19)
		Hemiplegia or paraplegia	10,097	1.4	101,623	1.4	0.95 (0.93, 0.97)
		Metastatic solid tumour	17,522	2.4	160,371	2.2	1.06 (1.05, 1.08)
		Mild liver disease	15,922	2.1	142,440	2.0	1.09 (1.07, 1.11)
		Moderate or severe liver disease	5,612	0.8	49,966	0.7	1.10 (1.07, 1.13)
		Myocardial infarction	61,985	8.3	522,655	7.2	1.14 (1.13, 1.15)
		Peptic ulcer disease	55,733	7.5	487,815	6.8	1.10 (1.09, 1.11)
		Peripheral vascular disease	52,918	7.1	457,310	6.3	1.11 (1.10, 1.12)
		Renal disease	163,397	22.0	1,396,081	19.3	1.12 (1.11, 1.12)
		Rheumatic disease	63,273	8.5	540,088	7.5	1.13 (1.12, 1.14)
	Antiviral history (at baseline)		81,419	11.0	716,244	9.9	1.10 (1.10, 1.11)

**Table 2:** Hazard ratio of developing dementia according to zoster vaccination

Vaccine type	Demographic	Category	Dementia type	Model 1: HR (95%C.I.)	Model 2: HR (95%C.I.)	Model 3: HR (95%C.I.)	E-value^a^
Zoster	All		All	0.80 (0.79–0.81)	0.78 (0.77–0.79)	0.78 (0.77–0.79)	1.89 (1.85)
			Alzheimer’s	0.94 (0.92–0.96)	0.91 (0.89–0.92)	0.91 (0.89–0.92)	1.44 (1.38)
			Other	0.73 (0.72–0.74)	0.71 (0.69–0.72)	0.71 (0.69–0.72)	2.19 (2.13)
	Gender	Male	All	0.86 (0.85–0.88)	0.83 (0.81–0.85)	0.83 (0.81–0.85)	1.71 (1.65)
			Alzheimer’s	1.04 (1.00–1.07)	0.99 (0.96–1.02)	0.99 (0.96–1.02)	1.12 (1.00)
			Other	0.79 (0.77–0.80)	0.76 (0.74–0.77)	0.76 (0.74–0.77)	1.98 (1.90)
		Female	All	0.76 (0.74–0.77)	0.74 (0.73–0.75)	0.74 (0.73–0.75)	2.05 (1.99)
			Alzheimer’s	0.87 (0.85–0.90)	0.85 (0.83–0.88)	0.85 (0.83–0.88)	1.62 (1.54)
			Other	0.68 (0.67–0.70)	0.66 (0.65–0.68)	0.66 (0.65–0.68)	2.38 (2.30)
	Age	70–74	All	0.86 (0.84–0.88)	0.81 (0.79–0.83)	0.81 (0.79–0.83)	1.76 (1.69)
			Alzheimer’s	1.02 (0.98–1.06)	0.97 (0.93–1)	0.97 (0.93–1)	1.23 (1.00)
			Other	0.77 (0.74–0.79)	0.72 (0.7–0.75)	0.72 (0.7–0.75)	2.12 (2.02)
		75–79	All	0.77 (0.76–0.79)	0.75 (0.74–0.77)	0.75 (0.74–0.77)	1.99 (1.94)
			Alzheimer’s	0.90 (0.87–0.92)	0.87 (0.85–0.9)	0.87 (0.85–0.9)	1.55 (1.46)
			Other	0.70 (0.69–0.72)	0.68 (0.66–0.7)	0.68 (0.66–0.7)	2.30 (2.22)
		80–84	All	0.82 (0.79–0.84)	0.8 (0.78–0.82)	0.8 (0.78–0.82)	1.81 (1.72)
			Alzheimer’s	0.93 (0.88–0.97)	0.91 (0.87–0.95)	0.91 (0.87–0.95)	1.44 (1.28)
			Other	0.76 (0.73–0.79)	0.74 (0.71–0.77)	0.74 (0.71–0.77)	2.03 (1.91)
		85–89	All	0.78 (0.62–0.99)	0.78 (0.61–0.99)	0.77 (0.61–0.99)	1.90 (1.13)
			Alzheimer’s	0.91 (0.60–1.38)	0.93 (0.6–1.43)	0.93 (0.6–1.43)	1.36 (1.00)
			Other	0.73 (0.55–0.98)	0.72 (0.54–0.96)	0.71 (0.53–0.96)	2.16 (1.27)
		90 +	All	0.83 (0.57–1.20)	0.74 (0.51–1.09)	0.75 (0.51–1.09)	2.02 (1.00)
			Alzheimer’s	0.57 (0.22–1.44)	0.39 (0.13–1.22)	0.39 (0.13–1.22)	4.52 (1.00)
			Other	0.90 (0.60–1.34)	0.83 (0.55–1.27)	0.83 (0.55–1.27)	1.69 (1.00)

Model 1: Adjusted for gender, age, and practice-level factors. Model 2: Further adjustments for IMD, ethnicity, and time-dependent indicators for the conditions in the Charlson Comorbidity Index. Model 3: Further adjustments for IMD, ethnicity, time-dependent indicators for the conditions in the Charlson Comorbidity Index, and time-dependent antiviral status

^a^E-value and minimum effect (confidence interval closest to the null) based on HR estimates with model 3

In the influenza vaccination cohort (Table 3), the results from the fully adjusted model (model 3) also suggested a slightly reduced hazard ratio (HR) risk of AD and other types of dementia (HR 0.96 with 95% of 0.94–0.97 and HR 0.89 with 95% CI of 0.87–0.90, respectively) but not with AD (HR 1.10 with 95% CI of 1.07–1.12). Results similar to those reported in zoster were seen with gender. In age subgroup analyses, results were inconsistent as reported in zoster vaccination. Moreover, non-significant HRs were observed in age group 70–74, 75–79 and 90 + .

**Table 3:** Hazard ratio of developing dementia according to influenza vaccination

Vaccine type	Demographic	Category	Dementia type	Model 1: HR (95%C.I.)	Model 2: HR (95%C.I.)	Model 3: HR (95%C.I.)	E-value^a^
Influenza	All		All	1.03 (1.02–1.05)	0.96 (0.94–0.97)	0.96 (0.94–0.97)	1.26 (1.20)
			Alzheimer’s	1.15 (1.12–1.18)	1.1 (1.07–1.12)	1.10 (1.07–1.12)	1.42 (1.33)
			Other	0.98 (0.96–1.00)	0.89 (0.87–0.91)	0.89 (0.87–0.91)	1.50 (1.44)
	Gender	Male	All	1.07 (1.04–1.09)	0.97 (0.94–0.99)	0.97 (0.94–0.99)	1.23 (1.11)
			Alzheimer’s	1.20 (1.15–1.25)	1.11 (1.07–1.16)	1.11 (1.07–1.16)	1.47 (1.33)
			Other	1.01 (0.98–1.04)	0.9 (0.88–0.93)	0.90 (0.88–0.93)	1.45 (1.35)
		Female	All	1.01 (0.99–1.03)	0.95 (0.93–0.97)	0.95 (0.93–0.97)	1.29 (1.21)
			Alzheimer’s	1.13 (1.09–1.17)	1.08 (1.05–1.12)	1.08 (1.05–1.12)	1.38 (1.27)
			Other	0.96 (0.93–0.98)	0.88 (0.86–0.9)	0.88 (0.86–0.9)	1.53 (1.46)
	Age	70–74	All	1.11 (1.07–1.16)	1.01 (0.97–1.05)	1.01 (0.97–1.05)	1.09 (1.00)
			Alzheimer’s	1.27 (1.20–1.35)	1.18 (1.11–1.26)	1.18 (1.11–1.26)	1.65 (1.46)
			Other	1.02 (0.97–1.08)	0.9 (0.85–0.96)	0.9 (0.85–0.95)	1.45 (1.27)
		75–79	All	1.06 (1.03–1.10)	0.98 (0.94–1.01)	0.97 (0.94–1.01)	1.19 (1.00)
			Alzheimer’s	1.14 (1.08–1.20)	1.07 (1.02–1.13)	1.07 (1.02–1.13)	1.34 (1.14)
			Other	1.01 (0.97–1.06)	0.92 (0.88–0.96)	0.92 (0.88–0.96)	1.41 (1.26)
		80–84	All	1.00 (0.97–1.03)	0.94 (0.91–0.97)	0.94 (0.91–0.97)	1.31 (1.20)
			Alzheimer’s	1.15 (1.09–1.2)	1.11 (1.06–1.17)	1.11 (1.06–1.17)	1.47 (1.32)
			Other	0.93 (0.89–0.96)	0.86 (0.83–0.89)	0.86 (0.83–0.89)	1.60 (1.49)
		85–89	All	0.97 (0.94–1.01)	0.91 (0.88–0.94)	0.91 (0.88–0.94)	1.44 (1.33)
			Alzheimer’s	1.08 (1.02–1.14)	1.03 (0.97–1.09)	1.03 (0.97–1.09)	1.21 (1.00)
			Other	0.93 (0.9–0.97)	0.85 (0.82–0.89)	0.85 (0.82–0.89)	1.62 (1.50)
		90 +	All	1.09 (1.04–1.14)	1.01 (0.97–1.06)	1.01 (0.97–1.06)	1.12 (1.00)
			Alzheimer’s	1.19 (1.09–1.3)	1.14 (1.04–1.25)	1.14 (1.04–1.25)	1.54 (1.25)
			Other	1.06 (1.01–1.11)	0.98 (0.93–1.03)	0.98 (0.93–1.03)	1.19 (1.00)

Model 1: Adjusted for gender, age, and practice-level factors. Model 2: Further adjustments for IMD, ethnicity, and time-dependent indicators for the conditions in the Charlson Comorbidity Index. Model 3: Further adjustments for IMD, ethnicity, time-dependent indicators for the conditions in the Charlson Comorbidity Index, and time-dependent antiviral status

^a^E-value and minimum effect (confidence interval closest to the null) based on HR estimates with model 3

In the zoster cohort, the smallest E-value was seen in AD (1.44 with E-value closet to 1 equal to 1.38). Similar patterns were also applied to stratified analyses by gender and age group (Table 2). In the influenza cohort, a smaller E-value was found (1.26 with E-value closet to 1 of 1.20) (Table 3).

Table 4 showed results of HRs of developing dementia according to zoster and influenza vaccination from the sensitivity analysis for the left censoring at 2 years of follow-up. HRs for all models showed similar pattern although slightly attenuated.

**Table 4:** Hazard ratio of developing dementia according to zoster and influenza vaccination—sensitivity analysis with left-censoring at 2 years of follow-up

Vaccine type	Demographic	Category	Dementia type	Vaccinated	Matched comparators	Model 1: HR (95% CI)	Model 2: HR (95% CI)	Model 3: HR (95% CI)
zoster	All		All	16,782	94,144	0.86 (0.85, 0.88)	0.83 (0.81, 0.84)	0.83 (0.81, 0.84)
			Alzheimer’s	6893	33,133	1.01 (0.99, 1.04)	0.98 (0.95, 1.01)	0.98 (0.95, 1.01)
			Other	9889	61,011	0.78 (0.76, 0.80)	0.75 (0.73, 0.76)	0.75 (0.73, 0.76)
	Gender	Male	All	7930	39,710	0.93 (0.91, 0.95)	0.88 (0.86, 0.91)	0.88 (0.86, 0.91)
			Alzheimer’s	2995	12,551	1.12 (1.08, 1.17)	1.07 (1.02, 1.12)	1.07 (1.02, 1.12)
			Other	4935	27,159	0.84 (0.81, 0.87)	0.80 (0.77, 0.83)	0.80 (0.77, 0.83)
		Female	All	8852	54,434	0.81 (0.79, 0.83)	0.79 (0.77, 0.81)	0.79 (0.77, 0.81)
			Alzheimer’s	3898	20,582	0.95 (0.91, 0.98)	0.92 (0.89, 0.95)	0.92 (0.89, 0.95)
			Other	4954	33,852	0.73 (0.71, 0.76)	0.70 (0.68, 0.73)	0.70 (0.68, 0.73)
	Age	70–74	All	4853	22,594	0.90 (0.87, 0.93)	0.84 (0.82, 0.87)	0.84 (0.82, 0.87)
			Alzheimer’s	2015	8099	1.08 (1.02, 1.13)	1.01 (0.96, 1.07)	1.01 (0.96, 1.07)
			Other	2838	14,495	0.81 (0.78, 0.84)	0.75 (0.71, 0.78)	0.75 (0.71, 0.78)
		75–79	All	8491	38,608	0.84 (0.81, 0.86)	0.80 (0.78, 0.82)	0.80 (0.78, 0.82)
			Alzheimer’s	3486	13,425	0.99 (0.95, 1.03)	0.96 (0.92, 1.00)	0.96 (0.92, 1.00)
			Other	5005	25,183	0.75 (0.73, 0.78)	0.72 (0.70, 0.75)	0.72 (0.70, 0.75)
		80–84	All	3377	22,939	0.88 (0.85, 0.91)	0.86 (0.83, 0.89)	0.86 (0.83, 0.89)
			Alzheimer’s	1369	8200	0.99 (0.93, 1.05)	0.97 (0.91, 1.03)	0.97 (0.91, 1.03)
			Other	2008	14,739	0.82 (0.78, 0.86)	0.80 (0.76, 0.84)	0.80 (0.76, 0.84)
		85–89	All	53	347	0.95 (0.70, 1.28)	0.95 (0.69, 1.31)	0.94 (0.68, 1.30)
			Alzheimer’s	21	86	1.37 (0.81, 2.31)	1.46 (0.82, 2.59)	1.54 (0.86, 2.75)
			Other	32	261	0.80 (0.55, 1.17)	0.77 (0.52, 1.15)	0.75 (0.50, 1.13)
		90 +	All	8	9656	0.40 (0.18, 0.89)	0.33 (0.15, 0.75)	0.33 (0.15, 0.75)
			Alzheimer’s	< 5	3323	0.59 (0.13, 2.67)	0.53 (0.12, 2.39)	0.53 (0.12, 2.39)
			Other	6	6333	0.36 (0.14, 0.91)	0.29 (0.11, 0.74)	0.28 (0.11, 0.74)
Influenza	All		All	7786	32,414	1.13 (1.10, 1.17)	1.06 (1.03, 1.09)	1.06 (1.03, 1.09)
			Alzheimer’s	2823	10,971	1.26 (1.21, 1.33)	1.20 (1.14, 1.25)	1.20 (1.14, 1.25)
			Other	4963	21,443	1.07 (1.03, 1.11)	0.98 (0.95, 1.02)	0.99 (0.95, 1.02)
	Gender	Male	All	3187	12,010	1.20 (1.15, 1.26)	1.09 (1.04, 1.15)	1.09 (1.04, 1.15)
			Alzheimer’s	1099	3963	1.32 (1.22, 1.43)	1.22 (1.13, 1.32)	1.22 (1.13, 1.32)
			Other	2088	8047	1.15 (1.08, 1.21)	1.05 (0.99, 1.11)	1.05 (0.99, 1.11)
		Female	All	4599	20,404	1.09 (1.05, 1.13)	1.03 (0.99, 1.07)	1.03 (0.99, 1.07)
			Alzheimer’s	1724	7008	1.23 (1.16, 1.31)	1.18 (1.11, 1.26)	1.18 (1.11, 1.26)
			Other	2875	13,396	1.02 (0.97, 1.07)	0.95 (0.90, 0.99)	0.95 (0.90, 0.99)
	Age	70–74	All	1440	5375	1.21 (1.14, 1.29)	1.12 (1.05, 1.19)	1.12 (1.05, 1.19)
			Alzheimer’s	594	1967	1.46 (1.32, 1.61)	1.36 (1.23, 1.51)	1.37 (1.24, 1.51)
			Other	846	3408	1.08 (1.00, 1.17)	0.99 (0.91, 1.08)	0.99 (0.90, 1.08)
		75–79	All	1987	7327	1.12 (1.06, 1.18)	1.04 (0.98, 1.10)	1.04 (0.98, 1.10)
			Alzheimer’s	790	2734	1.20 (1.10, 1.31)	1.16 (1.06, 1.26)	1.16 (1.06, 1.26)
			Other	1197	4593	1.07 (1.00, 1.14)	0.96 (0.89, 1.04)	0.96 (0.89, 1.04)
		80–84	All	2208	7341	1.12 (1.06, 1.18)	1.06 (1.00, 1.12)	1.06 (1.00, 1.12)
			Alzheimer’s	843	2589	1.26 (1.16, 1.38)	1.22 (1.12, 1.33)	1.22 (1.12, 1.33)
			Other	1365	4752	1.04 (0.98, 1.12)	0.98 (0.91, 1.05)	0.98 (0.92, 1.06)
		85–89	All	1462	4502	1.07 (1.00, 1.15)	1.00 (0.93, 1.07)	1.00 (0.93, 1.07)
			Alzheimer’s	447	1378	1.10 (0.97, 1.24)	1.04 (0.92, 1.18)	1.04 (0.92, 1.18)
			Other	1015	3124	1.06 (0.98, 1.15)	0.97 (0.89, 1.06)	0.97 (0.89, 1.06)
		90 +	All	689	7869	1.19 (1.07, 1.33)	1.13 (1.01, 1.27)	1.13 (1.01, 1.27)
			Alzheimer’s	149	2303	1.42 (1.13, 1.79)	1.37 (1.09, 1.73)	1.37 (1.08, 1.72)
			Other	540	5566	1.13 (1.00, 1.28)	1.09 (0.95, 1.24)	1.09 (0.95, 1.24)

Model 1: Adjusted for gender, age, and practice-level factors. Model 2: Further adjustments for IMD, ethnicity, and time-dependent indicators for the conditions in the Charlson Comorbidity Index. Model 3: Further adjustments for IMD, ethnicity, time-dependent indicators for the conditions in the Charlson Comorbidity Index, and time-dependent antiviral status

## Discussion

In this study, we investigated the effect of zoster vaccination on dementia risk in a large UK population-based data, the CPRD. We reported an inverse association between zoster vaccination and dementia outcome in our fully adjusted model (HR 0.78, 95% CI: 0.77–0.79). For Alzheimer’s disease, the effect size is smaller (HR 0.91, 95% CI: 0.89–0.92) however this result is likely only significant because of the very large sample size involved. The magnitude is negligible. To investigate if the result seen was exclusive to HZ vaccination, we also explored the effect of influenza vaccine and dementia/Alzheimer’s disease. We found a slight decreased hazard risk with HR of 0.96 (95% CI: 0.94–0.97) for dementia and HR of 1.10 (95% CI: 1.07–1.12) for Alzheimer’s disease.

A number of population-based studies have suggested a link between herpes zoster infection or vaccine against shingles and dementia [6–9, 27]. There are, however, other population based-studies that did not show any association between zoster infection and dementia risk [28, 29].

To prevent viral HZ, HZ vaccine stimulates the body’s immune system such that when VZV reactivates, the immune system rapidly recognises it as harmful and will trigger a response to limit the infection.

Our findings are in keeping with other population-based studies. These studies were included in a recent meta-analysis study which was published in 2022 [16]. The study included 17 high quality population-based studies with 1,857,134 participants. The studies investigated various types of vaccine including vaccines targeting HZ. The results suggested a significant decreased risk of dementia by 31% in their subgroup analysis of HZ-vaccinated compared to non-vaccinated subjects. The author reported significant heterogeneity of the included studies; nevertheless, all studies showed a protective effect of HZ vaccination (HR 0.69, 95% CI: 0.67–0.72, *P* < 0.001). Our study reported a slightly smaller effect of risk reduction. Another study investigated the association of shingles vaccination with incident dementia in Wales between 2013 and 2020 using retrospectively collected national health data. The study showed adjusted hazard ratio: 0.72; 95% CI: 0.69–0.75). The authors suggested selection bias could account for the reduced risk [30]. In contrast, the study using CPRD (both *Gold* and *Aurum dataset*) with a population aged ≥ 50 years during 1988–2018 suggested no association between shingles vaccination and dementia (OR 0.95 with 95%CI 0.92–0.98). The fact that shingles vaccination is available in the UK for people aged 70 onwards, by inclusion of people aged 50 could reduce the overall estimated risk. The authors also suggested frailty may also play a role as the study had limited access to data to enable assessment of the frailty of the participants [31]. This was also the case for our study.

Our study derives strength from using a very large UK population-based dataset, CPRD *Aurum*. The CPRD collects fully coded patient electronic health records from GP practices. The CPRD data are available for research with an approved project. Our analyses accounted for confounding factors that other studies did not adjust for such as time-dependent viral medication and CCI. We explored our fitted model with fixed covariates and time-dependent covariates. The latter approach was recommended based on the assumption that factors may change over the period of time that the subject is observed [32]. The observed association between outcome and time-dependent covariates may be biased if they are not appropriately modelled [33]. In our study, anti-viral treatments and co-morbidity were the two factors that could potentially change over time, therefore fitting the model with these two time-dependent variables could reduce any bias in our findings. Model 1 (fitted with confounding factors including gender, age, and practice-level factors), model 2 (with further adjustments for IMD, ethnicity, and time-dependent indicators for the conditions in the Charlson Comorbidity Index-CCI) and model 3 (with further adjustments for IMD, ethnicity, time-dependent indicators for the conditions in the Charlson Comorbidity Index, and time-dependent antiviral status) results showed similar effects. In this study, we applied an index with an adjustment on indicators for each of the conditions to derive a CCI. Most of the studies in the literature used a combination of selected illnesses. CCI provides a more overview of health status. We also investigated the prevalence ratios of co-morbidity, which were similar between the two cohorts indicating that the potential for selection bias was low.

To enable comparison between zoster and influenza vaccination, we restricted both cohorts for eligibility from year 2013 onwards when zoster vaccination was widely available from the National Health Services. We investigated if a common vaccine such as influenza, when used over the same time period, had a similar effect to zoster vaccine*,* thereby testing if any vaccine, regardless of its type, showed similar effects. The analysis of influenza vaccination was carried out as a “comparator group” which aimed to help identify and resolve whether there was possible residual confounding [34]. Our results showed that influenza vaccination was also associated with reduction of dementia risk: however the effect was very small (HR 0.96 with 95% C.I 0.94–0.97). Influenza vaccine is routinely offered by the UK National Health Service to adult aged 50 and over and anyone with specific underlying health problems (https://www.nhs.uk/conditions/vaccinations/flu-influenza-vaccine/). Our findings were in-keeping with findings from recent meta-analysis studies. In January 2022, a meta-analysis of influenza vaccination by Veronese et al. reported a significant reduction associated with influenza vaccination and dementia risk (RR = 0.97; 95% CI: 0.94–1.00; *I*^*2*^ = 99%). The authors included 5 studies with 292,157 older people free from dementia at baseline. Each study adjusted for various confounding factors, however none of these studies adjusted for anti-viral treatment and co-morbidity adjustment was different between studies. The authors discussed potential bias introduced by not including the influenza vaccination status during follow-up as a covariate, except in one study [35]. Publication bias was also identified as a source of bias in their meta-analysis. In May 2022, another meta-analysis study investigated various types of vaccines and dementia risk. They reported significant risk reduction in all vaccinations including rabies (HR = 0.43), tetanus & diphtheria & pertussis (HR = 0.69), herpes zoster (HR = 0.69), influenza (HR = 0.74), hepatitis A (HR = 0.78), typhoid (HR = 0.80), and hepatitis B (HR = 0.82) vaccinations [16]. However, the study found that any individuals who received between 1 to 3 influenza vaccinations during the follow-up period had no reduced risk of dementia compared to unvaccinated individuals. Only individuals with at least 4 annual influenza vaccinations had a 49% reduced hazard risk of dementia (HR = 0.51, 95% CI: 0.32–0.80, *P* = 0.003). The authors addressed unmeasured or residual confounding as a potential bias of the analyses in the original studies. Despite the slightly decreased HR seen in our study, the benefit of influenza vaccine in preventing influenza itself is paramount, particularly in the elderly population.

We also performed an E-value analysis. This particular analysis is proposed as a sensitivity analysis in observational studies to assess the robustness of an association which has potential unmeasured confounders which could potentially bias the results. A large E-value implies that considerable unmeasured confounding would be needed to explain an effect estimate. A small E-value implies little unmeasured confounding would be needed to explain an effect estimate [26]. The E-value results for zoster vaccination using the HR from model 3 produced a value of 1.89 with a lower limit of the confidence interval of 1.85. This indicates that our finding for zoster vaccination of a reduced HR of 0.78 (95%CI 0.77–0.79) is therefore unlikely to be accounted for by unmeasured confounding factors. The result seen for the E-value for the influenza vaccine result (E-value 1.26 with E-value closet to 1 of 1.20), however, suggests that residual confounding could account for the magnitude of the result (0.96 with 95%CI 0.94–0.97)).

To explore if vaccination could directly influence dementia development over longer time periods after vaccination, we carried out sensitivity analysis with left-censoring at 2 years after vaccination (or matching for the comparators). Although slightly attenuated, the pattern of results remains the same.

Our results support the suggestion that there is a specific effect of vaccinations against Herpes zoster rather than a non-specific immune stimulatory effect of other types of vaccinations, given that the influenza vaccination did not show a large effect.

The limitations of our study included potential variation in the definition and use of medical codes between practices [36]. However, we included only practices that were classed as ‘research standard’. In addition, the records of anti-viral treatment could be incomplete due to missing information on over-the-counter medications. Furthermore, selection bias cannot be ruled out. For example, there is a possibility that people who had received vaccinations were more likely to visit a physician than those in the non-vaccinated group, potentially indirectly increasing the chance of dementia detection. We have adjusted for co-morbidity but as for any epidemiological study there remains potential for bias and confounding. For studies of the effect of vaccination, this may pertain in particular to the possibility that the group that takes up vaccination are inherently healthier [37]. In reality, the only way to fully address this in future studies is to undertake a randomised control trial.

## Conclusion

In conclusion, our study found a significant reduced hazard of developing dementia in individuals who had had HZ vaccination as compared to non-vaccinated individuals.

### Supplementary Information

**Additional file 1.** Product codes for herpes zoster vaccine in Aurum database. The file listed product codes for herpes zoster vaccine used in Aurum database.

**Additional file 2.** Medical codes for herpes zoster vaccination in Aurum database. The file listed medical codes for herpes zoster vaccination used in Aurum database.

**Additional file 3.** The distribution of the number of matched comparators – by matched set. The file depicted number and percentage of matched comparators.

**Additional file 4.** Product codes for influenza vaccine in Aurum database. The file listed product codes for influenza vaccine used in Aurum database.

**Additional file 5.** Medical codes for influenza vaccination in Aurum database.  The file listed medical codes for influenza vaccination used in Aurum database.

**Additional file 6.** Medical codes for dementia in Aurum database. The file listed medical codes for dementia outcome in Aurum database.

**Additional file 7.** International Classification of Diseases 10th Revision (ICD-10) codes for dementia. The file listed ICD-10 codes for dementia outcome.

**Additional file 8.** Medical codes for Charlson Comorbidity Index in Aurum database. The file listed medical codes for Charlson Comorbidity Index used in Aurum database.

**Additional file 9.** International Classification of Diseases codes for Charlson Comorbidity Index in Aurum database. The file listed ICD-10 codes for Charlson Comorbidity Index used in Aurum database.

**Additional file 10.** Product codes for anti-viral medications in Aurum database. The file listed product codes for anti-viral medications in Aurum database.

**Additional file 11.** Codes for ethnicity in Aurum database.  The file listed codes for ethnicity.

**Additional file 12.** The numbers and rates of people developing dementia in zoster vaccinated and unvaccinated cohorts. The file depicted numbers and rates per 1000 year of people developing dementia in zoster vaccinated and unvaccinated cohorts.

## Data Availability

The data that support the findings of this study are from CPRD (https://cprd.com/) which were used under license for the current study, and so are not publicly available. Researchers would need to apply directly to CPRD, have a protocol approved and receive data directly from CPRD.

## Abbreviations

CPRD: The Clinical Practice Research Datalink
HES: Hospital episode statistics
ONS: Office for National Statistics
HR: Hazard ratio
95% CI: 95% Confident interval
HZ: Herpes zoster
VHA: Veterans Health Affairs
BRFSS: The Behavioral Risk Factor Surveillance System
MHRA: Medicines and Healthcare products Regulatory Agency
NHS: National Health Service
ICD-10: International Classification of Diseases
SNOMED: Systematized Nomenclature of Medicine
EMIS: Egton Medical Information Systems
CCI: Charlson Comorbidity Index
IMD: Indices of Multiple Deprivation
IQR: Interquartile Range
AD: Alzheimer’s disease
